# Professional roles in transformation through sharing rituals: a critical ethnographic study of Norwegian Recovery Colleges

**DOI:** 10.1186/s12913-025-12305-8

**Published:** 2025-03-07

**Authors:** Therese Ersvær Sjursæther, Christine Øye, Kristin Ådnøy Eriksen

**Affiliations:** 1 Faculty of Health and Social Sciences, Western Norway University of Applied Sciences, Inndalsveien 28, Bergen, 5063 Norway; 2 Faculty of Health and Social Sciences, Western Norway University of Applied Sciences, Klingenbergvegen 4, Stord, 5414 Norway; 3 Faculty of Health and Social Sciences, Western Norway University of Applied Sciences, Bjørnsonsgate 45, Haugesund, 5528 Norway

**Keywords:** Recovery colleges, Co-creation, Role transformations, Structure, Anti-structure, Sharing rituals, Facilitators, Mental health services

## Abstract

Recovery colleges (RCs) represent an innovation in mental health services. Facilitators with experiential knowledge or formal mental health training collaborate with students with lived experience or formal training to deliver courses in RCs. This paper examines the transformation of roles when co-creating new mental health services. We conducted a critical ethnographic study, collecting data from participatory observations in RCs at two locations and interviews with facilitators with formal mental health training and those with experiential knowledge. Drawing on “communitas” and “social ties,” we analyse the social dynamics of co-creation practices in RCs. Our findings reveal that traditional service roles and normative structures in mental health services are dissolved or redefined through these co-creation practices. Facilitators initiate sharing rituals that foster equality and communitas and continuously assess their disclosures. We understand this dissolution of the normative social structure as a liminal state in which facilitators gradually transition into new positions. However, significant differences in social status and the daily lives of facilitators and students challenge communitas, indicating that the dissolution of roles can be temporary. While co-creation practices in RCs demonstrate significant potential in transforming roles and enhancing mental health interventions, sustaining these changes in broader mental health services remains challenging. Further research is needed to explore the sustainability of these transformed roles and their integration into broader mental health services.

**Supplementary Information:**

The online version contains supplementary material available at 10.1186/s12913-025-12305-8.

## Introduction

Mental health services face the challenge of providing care and services that are efficient and responsive to the needs of their population [1]. In recent decades, researchers such as [2–9] have noted the need for greater knowledge and understanding of mental disorders as alternatives to dominant medical understanding and treatment. The need for change involves redefining service roles and increasing the influence of people with experiential knowledge that complements, challenges and expands professional roles and existing knowledge to improve services [10].

To address these challenges, municipalities in Norway have developed RCs since 2017, inspired by similar centres in England. These RCs supplement existing services and treatment for people with substance use and mental health challenges. They promote opportunities and the belief that people can live meaningful and satisfying lives, even with significant challenges in daily life. The RCs strengthen students’ connectedness, hope, identity, meaning, and empowerment through knowledge, reflection, and sharing experiences. These dimensions of recovery, referred to as CHIME, represent a framework for understanding and promoting recovery-oriented practice [11]. The RCs adopt an educational rather than a therapeutic model, allowing service users to take on new social roles as students [12].

Co-creation, a top priority in developing RCs [12], is an innovative process for addressing public tasks or social problems. It involves exchanging and applying diverse experiences, resources, competencies, and ideas from various individuals [13, 14]. In the context of RCs, co-creation brings together facilitators with experiential knowledge, students with lived experience, and facilitators and students with formal mental health training. Together, they design and deliver all aspects of the RCs [15]. The term lived experience in this context refers to the cumulative experiences that students have accumulated throughout their lives, particularly concerning substance use and mental health challenges [16]. Experiential competence is the active and conscious application of these personal experiences in a professional context, aiming to improve services or assist others [17]. The composition of the different types of experiences and backgrounds varies in different courses and settings.

RCs seek to achieve a strong community through co-creation and a sharing culture. A communication culture from one’s daily life appears essential for stimulating co-creation. The exchange of expertise and ideas about challenges, life skills, and contributions of both students and facilitators represents the knowledge and content of the course. Their way of practising co-creation challenges the traditional hierarchy through equal relationships, with a reduced distance and a ‘they and us’ distinction between facilitators and students [18].

To understand these co-creation processes, we draw on the theories of Victor Turner [19–22] and John Barnes [23], examining social dynamics and structural changes in RCs. Turner [19–22]**,** renowned for his contributions to symbolic and processual anthropology, developed the theory of liminality in rites of passage. He highlights three ritual stages: detachment, liminality, and reintegration. Detachment refers to the initial separation from the individual’s previous role or status, liminality is the transitional phase characterised by ambiguity and openness to new identities and roles, and reintegration marks the return to society with a new status or role. Turner focuses mainly on the experience of communitas—a deep sense of community wherein individuals achieve mutual meaning independent of societal hierarchies. In the context of our study, ‘communitas’ refers to the sense of unity and shared identity that emerges among facilitators and students in the co-creation process, transcending their roles and backgrounds. The co-creation process involves a series of rituals that facilitate the sharing of experiences, knowledge, and ideas. These rituals are crucial in breaking down traditional hierarchies and fostering a sense of communitas among students and facilitators.

While Turner concentrates on the profound community that arises in liminal stages, Barnes’ analysis offers insights beyond traditional class divisions, examining how individuals connect through kinship, friendship, and work relationships. His concept of ‘social ties’, which refers to these connections, is particularly relevant to our study [23]. It helps us understand the relationships and interactions among students and facilitators in the co-creation process and how social status and everyday life influence their roles in transformation.

## Background

Previous research acknowledges that co-creation is essential to RCs [1] without describing how facilitators and students practice the processes. Few empirical studies on RCs focus on roles and what co-creation entails in the course. There is a notable gap in the literature regarding the specific processes through which these roles are developed and enacted. While existing studies highlight the importance of co-creation and its impact on roles, they often lack detailed descriptions of the mechanisms and practices that lead to these role transformations. This paper aims to fill this gap by examining how facilitators and students in RCs actively engage in co-creation processes and how these interactions shape their roles.

Concerning the role of facilitators with formal mental health training, previous research has revealed that their participation in co-creation processes in RCs affects their perceptions of students and their passion and motivation at work. It involves changes in attitudes and professional practices, such as power dynamics, language, and information sharing [1]. The most reported change is a softening of established roles between facilitators and students [12]. Crowther and Taylor’s [12] study identified a beneficial impact on the well-being of facilitators in RCs, although the level of responsibility was problematic for some facilitators with experiential knowledge. Bester, McGlade and Darragh [24] determined that co-creation in RC positively affects professional attitudes and power dynamics between facilitators and students. Facilitators have adopted recovery-oriented practices and established equal relationships with students. Some facilitators now share their mental health challenges and feel greater empathy and less stigma towards students. Knowledge from formal training and experiential knowledge are equally valued. The articles in their study discussed the positive change in the balance of power. However, sometimes, facilitators with formal mental health training find adapting to their new roles as equals to students challenging [24]. Steen and Tuurnas [25] have studied how co-creation defines the role of facilitators with formal mental health training. They found implications for relational competence and facilitating and mobilising others. Furthermore, they highlighted the need for professionals to demonstrate an open attitude towards collaboration to motivate students to co-create.

Concerning the role of facilitators with experiential knowledge, Selbekk et al. [26] reported that these facilitators experienced opportunities to assume new positions in their recovery process. These transitions included moving from an institutional identity as “sick” to seeing themselves as “whole persons,” shifting from being recipients of care to taking active responsibility for their life changes and transitioning from isolation to becoming part of a supportive community.

This paper aims to examine the transformation of roles when co-creating new mental health practices. It intends to describe how facilitators in RCs facilitate co-creation processes during the delivery of courses in RCs and discusses the findings by considering the perspectives of Turner’s term of communitas [19] and the social ties of Barnes [23]. We consider structural and cultural conditions when answering the research question: *How do co-creation processes in Recovery Colleges alter the roles of facilitators considering the perspectives of communitas and social ties?*

## Methods

We conducted a critical ethnographic study, a methodological approach that challenges power relations and political inequality, highlighting social exclusion, marginalisation, and injustice [27, 28], which is appropriate since the research context involves changes in power relations, roles and services for people prone to social exclusion and marginalisation. Critical ethnography addresses structural, historical, and cultural conditions that are relevant to roles and how facilitators are affected by external factors [28].

The empirical foundation for this article is data from documents about RCs, participatory observations, focus groups, and in-depth interviews with facilitators with formal mental health training and facilitators with experiential knowledge at two different RC sites. Our more extensive study encompasses the entire course design and delivery spectrum, capturing facilitators’ and students’ viewpoints. This article examines a subset of the data concerning co-creation during course delivery to delve into the facilitator’s roles and how they change through co-creation processes.

As ethnographic researchers, we have spent time in the field and participated in two complete courses to develop an insider’s perspective on what is happening in RCs, gain trust, and foster close communication with facilitators and students. By observing and experiencing practice, we can discover changes in professional roles and reflect on what it means for co-creation and mental health services.

### Settings and participants

The courses were held at RCs located at two distinct sites in Norway. The first site was in a medium-sized municipality with 50,000 residents, and the second was in a cluster of municipalities with a combined population nearing 300,000. The table below provides a detailed overview of the settings, participants, and data collection methods (Table 1).

**Table 1:**
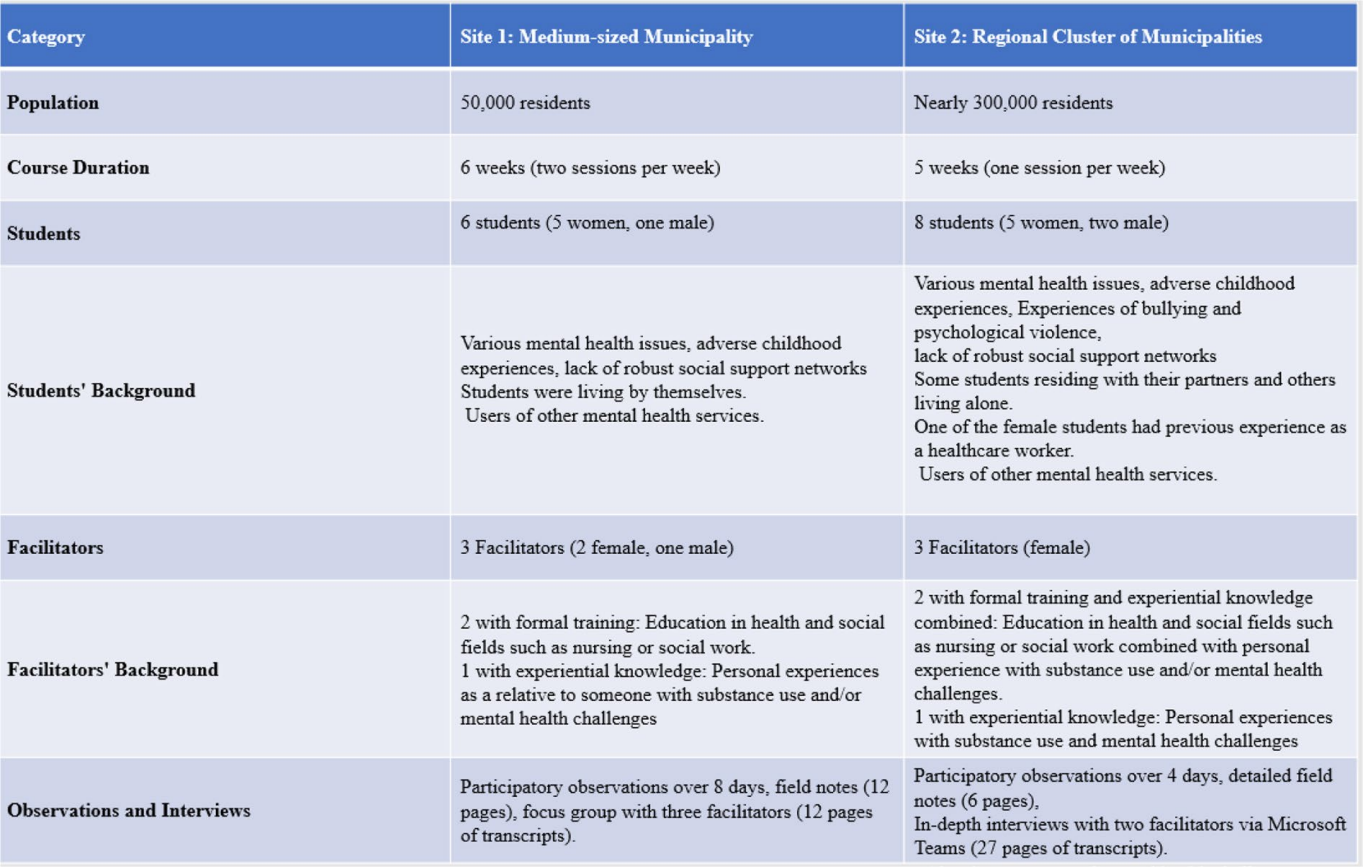
Overview of settings, participants and data collection methods

The first course, organised by municipal mental health services, had a structured format comprising two sessions per week over six weeks. The second course, operated by a nonprofit organisation funded by the County Governor and the Directorate of Health, spanned five weeks with weekly gatherings.

Among the facilitators, those with formal mental health training had educational backgrounds in health and social fields. Facilitators with experiential knowledge contributed their personal experiences with substance use and/or mental health challenges or their experiences in supporting relatives through similar issues.

The students from both sites faced a range of mental health challenges, adverse childhood experiences, and inadequate social support systems. Despite RCs being open to relatives and mental health service providers, the courses we examined were exclusively attended by students with lived experience [29], ensuring a focus on their unique perspectives.

The first author read 120 pages of RC documents in the initial data collection phase, including brochures, reports, and quality standards. These documents provided a foundation for subsequent participatory observations and interviews. Over 12 days, we documented detailed field notes while engaging with the course material alongside students. Post-course interviews included a focus group with three facilitators and in-depth interviews with two facilitators via Microsoft Teams, resulting in 40 pages of transcripts.

### Analysis

The first author’s background as a social worker with extensive experience in Norwegian mental health services influenced the research process. The researcher’s previous study about peer support workers, where the findings involved a more personal professional role [30], influenced her interest in sharing rituals and how the roles changed in the RCs. The researcher maintained critical self-awareness throughout the process, illuminating the researcher’s evolving positionality and impacting data collection and the presentation of findings [28]. This critical self-awareness involved continuous reflection on personal biases and assumptions, supported by frequent supervision sessions. During these sessions, the researcher discussed observations and interpretations with supervisors, which helped to identify and mitigate potential biases. This collaborative reflection ensured a more balanced and objective analysis, enhancing the credibility of the findings.

We analysed and interpreted data from observations and interviews using the three analytical levels referred to by Fangen [31]: first-degree, second-degree, and third-degree interpretations. (See Table 2 for a detailed description of each analytical level). Through this multilevel analysis, we aimed to contextualise and situate our observations within a broader theoretical context, providing a new perspective on the observed phenomena.

**Table 2:**
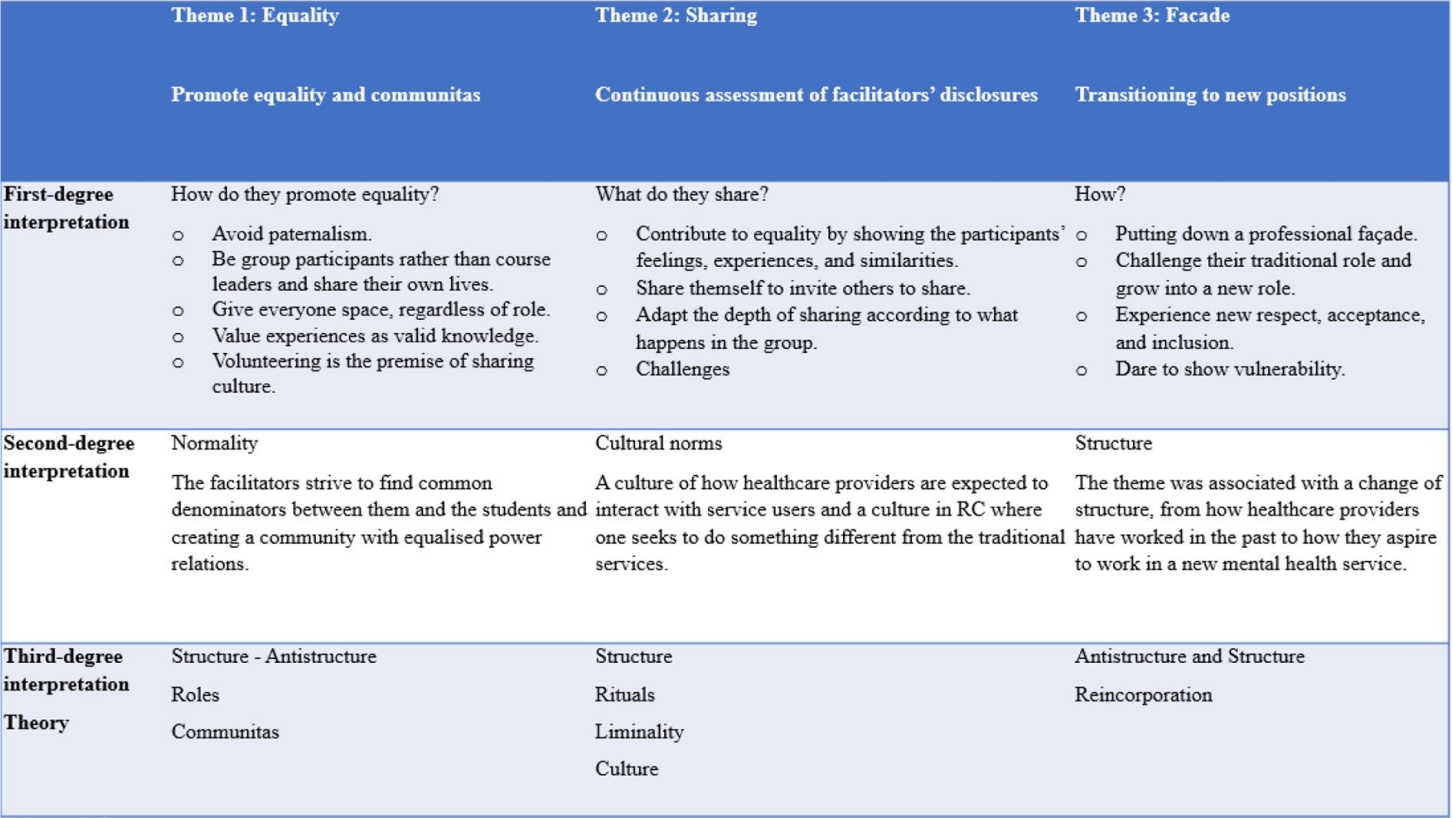
Analysis: first-degree, second-degree, and third-degree interpretations

By first-degree interpretation, we determined what we observed and interpreted the event with the concepts used by the study participants [31]. First, we read the field notes and interview transcripts line by line and noted common-sense categories. We divided the text into several parts and reorganised the data into thematic headings: ‘Promote equality in the group’, ‘Continuous assessment of facilitators’ disclosures’, and ‘Grow into new positions without a professional facade’.

In the second-degree interpretation, we transcended the participants’ immediate understanding [31]. This stage involved identifying patterns and themes within their experiences and contextualising these findings within broader contexts, such as the cultural and social frameworks relevant to mental health services. During this phase, ideas emerged from the data, leading us to identify ‘analytical threads’. These threads allowed us to delve deeper into the data and uncover new insights. We related the concept of equality to normality, which concerned the facilitators striving to find common denominators between them and the students and creating a feeling of community with equalised power relations. We linked the theme of ‘assessments of disclosing’ to cultural norms, specifically focusing on how healthcare providers should interact with service users and the desired culture in this new service. The theme of ‘growing into new positions’ was associated with a structural change, from how healthcare providers have worked in the past to how they aspire to work in a new mental health service. We interpreted the connection between these three themes as a role in change.

In third-degree interpretation, we were critical of the participants’ understanding and sought hidden agendas and needs. Without taking their views for granted, we critically interpreted them by drawing on different contexts of meaning [31]. Here, we used Turner’s theory [16–19], which involves rites of passage and communitas. We used the analysis to understand the transformation as an anti-structure, a dissolution of the normative social structure in traditional mental health services [19, p. 28]. The critical distinguishing features in the transformation of roles are detachment, liminality in rites of passage, and reincorporation. We used these characteristics of this process to present the data sorted in the final codes: ‘Promote equality and communitas’, ‘Continuous assessment of facilitators’ disclosures’ and ‘Transitioning to new positions.’ Barnes’ [23] theory about social ties was relevant to understanding how students in RCs interact. According to his theory, the social network is a system of ties between pairs of people arising from kinship, friendship, and acquaintance considerations. The connections are between people who regard each other as approximate social equals. Barnes refers to these relationships as class networks, including social activities such as mutual help, entertainment at home, and ties between people.

### Ethical considerations

We conducted the research with the utmost respect for ethical principles. Facilitators who participated in the interviews gave informed consent, and their anonymity was maintained by not distinguishing between the different sites. The RC is a vulnerable site of research where those involved share much from themselves and their private lives. We handled this sensitive information carefully and respected participants’ confidentiality by slightly re-formulating their statements. Moreover, all the participants gave informed consent, and we held up their right to withdraw from the study. To elicit participants’ voices, we upheld epistemic justice via multiple research methods, such as participatory observation, in-depth interviews and focus group interviews.

## Results

We collected a critical ethnographic study to explore: *How do co-creation processes in Recovery Colleges alter the role of facilitators considering the perspectives of communitas and social ties?* Through a three-level analytical interpretation by Fangen [31], we discovered three findings that represent a transformation of the professional role, directly addressing our research goal: (i) promote equality and communitas, (ii) continuous assessments of facilitators` disclosures, and (iii) transitioning to new positions.

### Promote equality and communitas

Despite the facilitator’s different backgrounds, they experienced equality in their positions and shared ownership of the course. They practice co-creation by securing both competencies at all stages, from planning and precautionary interviews to conducting lessons, and strive for an equal distribution of tasks. The courses include short sections on theory, individual reflection exercises, and enhanced dialogue in groups and plenary sessions. Illustrative pictures, videos, and poems are essential to evoke emotions, memories, and thoughts. One of the courses introduces concrete tools to build healthy relationships and create the desired community for each participant.

Facilitators strive to establish communitas in RCs, understood as an experience of community between a group of people when their lives together achieve an ordinary, mutual meaning [22]. They promote equality in the group by avoiding paternalistic attitudes. In one of the interviews, one of the facilitators with experiential knowledge explained, “We have been very aware that we should not perceive a top-down philosophy. There should be a course group, and it should participate equally. Yes, we are a group.” This statement indicates reciprocity between participants who initially had different roles, such as facilitators, researchers, and other mental health service staff (see Table 3, Column 1). They help each other by participating equally, regardless of their position, and giving each other mutual advantages. Such reciprocity implies sharing experiences of one’s life, feelings, and vulnerability, including personal values, goals, networks, and strengths, and supporting each other. Facilitators actively participate in lessons, disclosing their personal lives to promote equality and communitas. Students who are accustomed to receiving help now support other students, including those with formal mental health training.

**Table 3:**
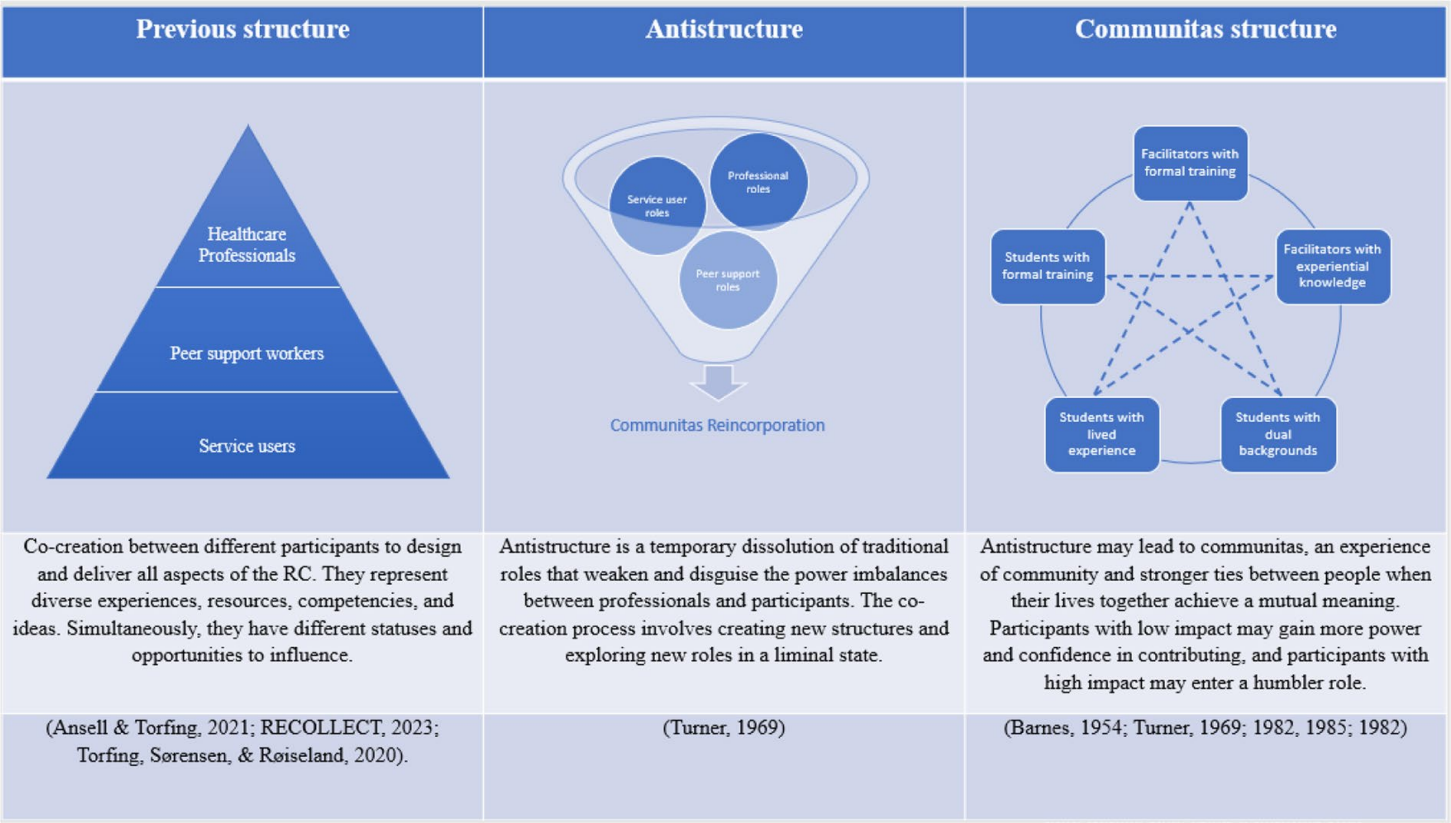
Analysis of co-creation rituals and the interplay of structure and anti-structure

Sharing rituals is essential to achieving communitas, as demonstrated in the field notes.

On the first day, the facilitators introduced us to the illustration pictures that would become a regular ritual. They placed cards with different motifs on the table before the lesson and invited everyone to find a card representing their identity. I chose a picture of a beach with two wooden chairs facing the sea, symbolising my love for the ocean. One of the facilitators chose a square to demonstrate that the person valued structure. Other students selected cards with monkeys embracing each other to illustrate their caring nature and a picture of nature to show their interest in outdoor activities. The last student chose an instrument indicating an interest in music and great talent with which we later became familiar. Once everyone had chosen their card, the facilitators invited us to share our thoughts. We ended with a structured round where the student and facilitators shared their reflections on their cards, allowing everyone to reveal a small part of their personal lives.

During the lessons, the facilitators organised various sharing rituals, such as reading poems, showing movies, performing exercises, and inviting everyone to share their reflections. They alternated between structured rounds where everyone shared their thoughts and open discussions for voluntary participation.

Facilitators also promote equality by giving everyone room and valuing experience as much as professional knowledge. Facilitators encourage participation in the group by reaching out to students and ensuring that their opinions are essential to the group. They highlight that the student’s inside is just as valid as what the facilitators present. One of the facilitators with formal mental health training explained:

The facilitators do not have the correct answer when we go through the CHIME elements; the answer comes from the group. I think it is also essential in practice to believe that the group possesses the process and the knowledge. We focus on their opinions, which are crucial for the group. What hope means for my colleague differs from what hope means for me. When the group realises their thoughts on hope are also important, it becomes easier for them to share. In a way, it defuses the sharing practice.

Sharing individual experiences and thoughts on a topic contributes to knowledge and learning. To encourage participation, they encouraged the students to share their experiences before facilitators shared their thoughts. One of the facilitators with formal mental health training shared the following: “After seeing a film, we are not the first to share our feelings, experiences, and impressions. We sit back, want input, and guide them on track before sharing ours.” In this way, the facilitators show that the information from the students is appreciated. In practice, we observed that the facilitators alternated between waiting for students’ contributions and modelling, where they shared their own experience first, for example, to demonstrate an exercise. Sometimes, the facilitators asked about the students’ thoughts and shared their own thoughts if none of the students volunteered. Active participation seems crucial in the course; students are encouraged to share, but it is entirely up to them what and how much they choose to present. The facilitators felt that the students were inspired by each other when they gradually shared their thoughts and that they had a positive influence on each other. One of the facilitators with formal mental health training said, “Eventually, people understood that it is nice to hear and be inspired by other people’s thoughts when people share. There is an influence there, and we influence each other very positively.” The quote shows the positive impact that sharing had on the group.

### Continuous assessment of facilitators’ disclosures

Facilitators acknowledged that it would inhibit the co-creation process if those with formal mental health training did not contribute when facilitating a culture of sharing. This acknowledgement forces them into a liminal state [19] of temporariness, vulnerability, and uncertainty, where they must continuously assess what they disclose to the group (see Table 3, Column 2). What facilitators choose to disclose about themselves depends on the group’s needs. One of the facilitators with experiential knowledge said:

I usually consider the group and how deep I can go when I share something. Sometimes, I can go much deeper because I see that someone is on the edge of sharing but is scared. Perhaps this lies in the integrated shame of childhood. Then, I can delve into my own experiences and say I have chosen to use this as a strength. In contrast, other times, I stay just below the surface and generally talk about things if the group is not ready to accept deeper sharing. You learn to read the group. I think there is a regulation of the group and the need, and then you balance the sharing accordingly.

Continuous assessments involve knowledge of shame, which can cause students to refuse to talk about their situation. When sharing, the facilitator can influence the students to communicate by going deeper into the facilitator’s own experiences. Some facilitators with formal mental health training emphasised sharing something meaningful so that other students did not perceive problems as superficial and minimised their concerns.

What could be problematic (…). It is as if they [the students] believe we as professionals do not have problems and portray my problems as simple. I shared some concerns about family life and children (…). Sometimes, I fear that something would hurt them if they did not have children or if they did not have that situation or family. You do not want to come across as somehow perfect, either. (…) After all, there is something about the balance of not sharing the deepest issues that you have not finished or processed yourself but also not sharing so easily that people think your life is so easy.

Facilitators with experiential knowledge favour the different roles. One said that she had never experienced students who perceived other people’s challenges as superficial. Professionals who are traditionally hidden behind a façade show more of who they are as a person, which has a significant effect and contributes to acceptance in the group. In practice, Facebook requests and examples from professionals included in the other students’ contributions demonstrated their acceptance.

I have never seen anyone comment or appear to have perceived something as shallow or too small. I think the setting is that a professional who used to be completely shut down now shares and shows that there is more to the façade. I think it has such a significant impact that there will be no measure of how much you share the truth and how your life appears.

In discussions about sharing goals that tend to differ from the lives of students and the fear that students experience professionals trying to rise above them, one of the facilitators with formal mental health training said:

As you said, I cannot share that I want to be a professor.’ There is something about our old professional point of view. They will not deal with the news that my good life is improving. However, everyone here can hear that my life is going well: I am rebuilding my house. None of them owns a home. One student could not pay for a bus ticket, and I am putting an extension on my house. It is a great contrast.

Facilitators from both backgrounds emphasised that the most important thing was that the contributions were genuine based on their experiences. Several facilitators avoided being too vulnerable and did not disclose personal stories or problems they had not processed. A facilitator with experiential knowledge described this as distinguishing the private from the personal. Everyone must consider what is okay to share and what they want to keep to themselves. Despite the confidentiality to which the students agree, one must assume that the information will leak. Therefore, it is essential to decide what information to provide others.

You should consider what you want to share (…). In other words, one has separated the personal and private and what one only wants to have for oneself that one can only share with a few. One has found the balance (…) because you have the moral duty of confidentiality, but some things will leak. Right? And then you are ready to hear it outside. Do not say anything that you do not want to hear back.

This facilitator disclosed information on a somewhat superficial level, without too much detail about painful experiences. For example, she could share bullying experiences without telling them details of events in detail. In this way, she avoided making herself too vulnerable.

I share, but I like to share superficially, not superficially, but how I have experienced things. However, I do not go into detail or share in a way that makes me vulnerable (…). For example, being bullied in an elementary school is not good. You are very insecure, and I have been insecure throughout my life. (…). I do not share anything more than that. I will not go into what has happened. Those who have experienced bullying know this. You do not have to go into detail. After all, it is recognisable to those who are supposed to recognise it.

Here, the facilitator provides information about her life without discussing trauma. A facilitator with formal mental health training described a challenging experience with a course in which several students with comorbid disorders competed to share the worst experiences of their personal stories. Thus, it became difficult for students with better functional abilities who chose to quit the course. As part of the liminal state, the facilitators agreed not to induce the students’ trauma and strove to highlight valuable strategies in their recovery process.

Experience of the past when it becomes a competition to share the worst. Then, others think, ‘You know what, we do not bother.’ Therefore, they do not share because they do not want to be part of that ‘worst carousel.’ It can be a problem for the sharing culture if the compositions are not good enough; then, you can get a dichotomy in the group that makes one part withdraw.

This example shows that poor group composition can cause some students to dominate sessions and others to become passive or drop out. The facilitators highlighted other challenges, such as when someone chose not to share or contribute to the course. Furthermore, the lack of volunteerism or participation under the experience of coercion from others was an inhibitor of co-creation and the culture of sharing.

### Transitioning to new positions

Facilitators with formal mental health training challenged their traditional role by promoting communitas and changing their understanding of their role in the liminal state. A professional façade is a high priority in ordinary mental health services, and facilitators said that employees could experience being seen as unprofessional when sharing information from their own lives. Now, they grow into new positions where they disclose their experiences, feelings, and vulnerability, and the professional façade becomes less prominent (see Table 3, Column 3). One of the facilitators with formal mental health training explained:

You have an experience that you should not share a piece of sh*t! We are professionals (…). Everything we say and do should be factual and sensible. When we share our personal feelings and thoughts, there is something that takes time to improve. Therefore, it takes time, but it is something to feel that you get acceptance in the group and that it is also okay to feel, and then you get acceptance from the group. (...) You are human, too. You are not just a professional.

The facilitators experienced through the liminal state that sharing led to great respect and acceptance from the students. They emphasised that stories of their life experiences could normalise problems, make students feel less alone with their issues, and give them a broader perspective. Given the relational gains of sharing and seeing that their contributions are helpful to people with substance use and mental health challenges, facilitators showed positive attitudes and enthusiasm to be more open to service users outside of the RC. One of the facilitators with formal mental health training shared how his professional role had changed.

I have a lower threshold to share. When they [service users] present their problem, I can share that I have the same or similar problem. It helps to normalise their situation. It is no longer as large, harmful, or heavy. More people are struggling with it. I work with them - the healthiest in society. There is something about it: When you feel like a crisis, you come in, and someone tells you that what you are experiencing is entirely normal. Only your emotions have run wild. In addition, when they understand that this is within what is okay, they manage to regulate themselves. Then, life goes much better. Moreover, knowing that the guy sitting here is not necessarily the best parent in the world either, who thinks it is nice to give the iPad to the child to get some extra time off on the weekends? Therefore, being able to share a little here lowers the threshold for daring to come here and accepting that things can improve.

This quote from this facilitator indicates that whether and what one discloses depends on the service recipient. The facilitator quoted above worked with well-functioning people who received a low-threshold offer from the municipality. Another facilitator with formal mental health training worked in a municipal service where users had comorbid disorders and poorer functioning abilities. Despite some difficulties, the person also saw gains from sharing:

I have some of the same experiences that I can share more. At the same time, I have the patient group, so you must consider who… I make judgments based on the person to whom I am speaking. However, I think you have something to gain from sharing, especially in relational terms. What I can share is very individual.

This quote indicates that despite their enthusiasm for what is happening in RCs, they still need to assess what they can disclose to service users of other mental health services and how to transfer what they have experienced and learned in RCs.

Facilitators with experiential knowledge develop their role by using their experience to help others. They transition from being students in courses to becoming healthcare providers. Initially insecure, they gradually take on more responsibility and see themselves as equal facilitators alongside their colleagues with formal mental health training. They described this transformation as challenging themselves and seeking support from their colleagues to find their role. One facilitator shared:

I would not be here today without Recovery College. The professionals there have met me as a human being and recognised my value. Often, the professionals who work at the college are outstanding—spacious, safe, and open, not instructive. I have met fantastic people through the college, which has made me who I am today. I would not have achieved this without them. With the safety and security I feel, I know I have value.

This tacit knowledge—gained through the hard school of life—provides a wealth of understanding you cannot read in books. You realise you possess enormous knowledge, and professionals help you articulate this. They help you see your many resources, which is very empowering. Additionally, you can offer a different perspective on theory, providing another angle.

These examples show that facilitators with formal mental health training and experiential knowledge undergo separate transformations to find new positions. These transformations occur through co-creation and the influence of people with different knowledge and backgrounds.

## Discussion

Previous research highlights the importance of co-creation and its impact on roles, mainly focusing on softening established positions [1, 12, 24]. Studies have shown that facilitators with formal mental health training change attitudes and professional practices, including power dynamics, language, and information sharing [1]. Crowther and Taylor [12] noted the beneficial impact on the well-being of facilitators despite some challenges faced by those with experiential knowledge, such as the level of responsibility. Bester, McGlade, and Darragh [24] further substantiated these findings, indicating that co-creation positively affects professional attitudes and power dynamics, leading to recovery-oriented practices and equal relationships between facilitators and students. Nonetheless, facilitators with formal mental health training sometimes find adapting to their new roles as equals to students challenging [24]. Steen and Tuurnas [25] emphasised that relational competence and an open attitude towards collaboration are crucial for effective co-creation.

In contrast, facilitators with experiential knowledge have found opportunities to assume new roles in their recovery process, moving from seeing themselves as “sick” to “whole persons” and transitioning from recipients of care to active contributors [26]. This transformation includes becoming part of a supportive community, highlighting the dynamic nature of their roles in co-creation environments. However, these studies lack detailed descriptions of the mechanisms and practices that lead to these role transformations.

In this study, we explored how co-creation processes in RCs alter the roles of facilitators, considering the perspectives of communitas and social ties. Our primary findings indicate a transformation of the professional role within RCs through three main mechanisms: (i) promotion of equality and communitas, (ii) continuous assessment of facilitators’ disclosure, and (iii) transitioning to new positions.

Facilitators in Norwegian RCs, with formal mental health training and those with experiential knowledge, foster co-creation by engendering an atmosphere of equality and communitas. They use language as a tool and invite everyone to share their experiences, regardless of their previous roles, positions, and power to impact (see Table 3, Column 1). Their relational competence helps mobilise and motivate students to engage in co-creation, contributing to the study by Steen and Tuurnas [25]. Facilitators continually assess the depth of what they disclose from their lives based on the group’s needs and boundaries. The facilitators eventually transition to new positions that differ significantly from those in traditional mental health services, focussing on education and learning rather than health transformations. Our findings exemplify how the transformation of roles occurs through different stages: detachment, liminality, and reincorporation [22].

Detachment represents a passage from an earlier stage regarding social structure and cultural conditions. Facilitators with formal mental health training detach themselves from the traditional professional role as they know it and from traditional expectations of holding a professional façade. Facilitators with formal mental health training change their mindset by not focusing on diagnosis, role sets, and status, except in their introduction, where they provide information about their background. Quotes such as “We are one group” and “We are all students” demonstrate that facilitators intentionally use their language to create and carry out a reality of equality and anti-structure, which clashes with the structure of traditional mental health services [28, p. 156]. Facilitators with formal mental health training do not consider themselves experts with more valid knowledge of the needs of service users, which is a characteristic of medical tradition.

During the liminal state, facilitators and students traverse a cultural realm with few or no attributes of the past or future state. Cultural scripts [19, 20] that dictate traditional expectations for healthcare providers’ conduct change in this liminal state. These facilitators are neither fully aligned with their past professional roles nor defined by future expectations. They are not strictly professionals, facilitators, or students but inhabit a transitional space between their prior roles or positions. This liminality presents a continuous challenge, prompting them to reassess best practices, such as determining the appropriate information to disclose within the group and fostering equal contributions from all students.

The anticipation of their roles in the liminal state remains unclear, and traditional expectations continue to influence facilitators’ interactions in ways that can be uncomfortable [20, 22], leaving them feeling unprofessional and uncertain about their roles. Co-creation practices become feasible only through the dissolution or transformation of these cultural scripts. Consequently, the group of students and facilitators contrasts with traditional society, which is structured, differentiated, and often hierarchical, distinguishing individuals in terms of higher and lower status [22]. Both facilitators and students grapple with the absence of a prescribed behaviour as roles remain in flow. Uncertainty surrounds the detachment from and expectations of traditional roles. Facilitators with formal mental health training find themselves tethered to their professional roles while simultaneously exploring new roles. This uncertainty may elucidate the findings of previous research indicating that facilitators with formal mental health training sometimes struggle to adapt to new positions as equals to service users [24].

Structural and cultural conditions demonstrate the complexity of finding a new role. Facilitators strive for equality, an essential value in Norwegian culture, which means treating others as equals, regardless of whether their income, education, interests, and occupation differ significantly from their own [23]. However, despite the core value of equality in RCs, students and facilitators vary according to economic, social, and cultural dynamics. Significant differences in their financial situation become apparent in the course. Facilitators, especially those with formal mental health training, typically have a stable income and generally live comfortable, middle-class lives with homes, children, and pets. Several students rely on welfare funding and live below the poverty line in Norway. These differences create entirely different living conditions. For instance, the facilitator who built his own house contrasts starkly with the participant who could not afford a bus ticket to attend the course. These facilitators must especially consider this social inequality in their ongoing assessments of what they share. This awareness of inequality aligns with Barnes’ [23] statement that people often hide their views on social inferiority, as it is inappropriate for anyone to show that they consider themselves superior openly.

Simultaneously, liminality allows one to introduce practices in line with new cultural scripts. Facilitators organise sharing rituals to establish communitas to show their feelings and life experiences and achieve an ordinary, mutual meaning (communitas) with students despite their differences. Drawing on Barnes’ theory, the liminal state and striving for equality and communitas are about establishing stronger ties between the students and facilitators. Together with other students, they experienced trials based on humility and homogeneity, which allowed them to establish a fellowship. Facilitators share information from their own lives to invite others to share and adapt the depth of communication based on what is happening in the group. This new practice invites students to recognise themselves in others, which is fundamental to experiencing connectedness. The new cultural script entails not going too deep into the bad experiences of their lives. They model behaviour and guide students not to leave too much room for trauma and prevent the development of what one facilitator referred to as the ‘worst carousel’—a situation where there is a competition to share the most traumatic experiences.

The roles are in flow, leading to tension among facilitators in RCs, as they feel uncertain about what they can share without offending students and hurting them about something they lack in life. Facilitators with formal mental health training try not to appear snobbish when discussing their life challenges and present themselves as people with faults and shortcomings. They strive for communitas to allow students with substance use and mental health challenges to feel less alienated. Accordingly, the liminal state enables the transformation of new roles for facilitators and students. In this way, the liminality of co-created practices can allow students to establish a higher status and a socially accepted role as active students.

In reincorporation*,* facilitators with formal mental health training have established a new role and a humbler status, with a less prominent professional facade, and appear more open in encounters with people with substance use and mental health challenges. The data show that they see the benefits of being more vulnerable and honest with service users, and the result of the RCs is that they have a lower threshold of sharing in their relationships with service users outside of the RCs. These findings align with previous studies that show how co-creation processes soften the professional role and influence attitudes and practices related to power dynamics, language, and information sharing [1, 12, 24]. Reincorporation into new roles also applies to facilitators with experiential knowledge, who may have developed a new understanding and approach to their role through participation in RCs. They actively work on utilising their competence in the co-creation process. This process contributes to transforming their lived experience with substance use and mental health challenges into valuable experiential knowledge. As a result, the healthcare provider’s role within the service is actively reshaped, emphasising the integration of lived experiences into professional practice.

Our findings clarify how co-creation processes within RCs can alter professional roles through sharing rituals. This alteration of professional roles involves the facilitation of co-creation and initiating and participating in sharing rituals. The role of healthcare providers in RCs differs significantly from traditional mental health services. In this context, healthcare providers can be individuals with formal mental health training, experiential knowledge, or a combination of both. This new professional role focuses on facilitation, leading learning processes that emphasise education and self-perception transformation rather than health transformation.

However, these transformations are not necessarily sustainable. The liminal state, characterised by equality and the dissolution of roles, appears to be temporary. At some point, we assume that facilitators and students return to services where different functions, such as service providers and service users, come into play. Nevertheless, it is worth considering whether the expectations of their roles change permanently and whether experiential knowledge gains greater importance over time. Barnes [23] reported that when members of a society perform their jobs, they are no longer equal. Chains of command and functional differentiation organise their jobs and positions. There is someone in charge who gives orders, and their subordinates obey. This fact aligns with Turner’s [19] statement that inclusiveness, the anti-structure of communities, is not permanent. According to him, it is impossible to eliminate roles, status, and order in a society. Eliminating the structure is also undesirable, as it regulates and preserves culture and provides safety to members. However, mitigating the existing structure’s negative aspects is appropriate [19]. The findings reveal facilitators’ positive attitudes towards the new cultural scripts emerging in RCs. Their attitudes suggest that the anti-structure they have worked to establish may reduce the negative aspects of previous structures in mental health services. Professionals have reincorporated a new role after experiencing anti-structure and communitas (see Table 3, boxes Columns 1 and 2), which they apply to their approaches in other mental health services. Sharing rituals might also be used outside RCs, enabling, and encouraging facilitators and students to assume new positions. If so, we could argue that co-creation practices can form a new structure that separates people, defines their differences, and limits their actions to a lesser extent. This new structure, characterised by a humbler professional role within health care, equality, communitas, and stronger ties between healthcare providers and service users, can help professionals gain acceptance and trust from service users. Hopefully, this will enable services to provide better mental health services that respond more effectively to the population’s needs by normalising problems, eliminating stigma, and providing people with substance use and mental health challenges with a broader perspective.

### Implications for practice

The transformation of roles, including detachment, liminality and reintegration, and awareness of inequality, may apply to other co-creation processes. Co-creation facilitators must distribute power to involve everyone in the co-creation process, regardless of their background. As observed in RCs, anti-structure is a temporary dissolution of traditional roles that weakens and disguises the power imbalances between facilitators and students. The co-creation process involves creating new structures and exploring new roles in a state of liminality and anti-structure. Experiencing an anti-structure promotes communitas, equality, and stronger ties among students. They could strive for mutual understanding and shared goals by dissolving roles and structure. In this study, we observed that students who traditionally had low impact gained more power and confidence in contributing. In contrast, those with a high impact adopted a humbler role.

Knowing that different students represent economic, social, and cultural variations is essential when practising co-creation. The findings show that the role of healthcare providers involves conscious judgments about what to disclose and the depth of sharing. Facilitators must demonstrate high-level professionalism, academic skills, and presence. They appear to be expanding their professional role so that their primary task becomes understanding how they can enable students to engage, be willing to engage and share on equal terms with students and understand what it entails to facilitate group processes in ways that enable growth for all.

When examining the transformation of roles in co-creation, it is necessary to consider the interplay between anti-structure and structure and understand the co-creation processes that require evolution into new roles.

### Strengths and limitations

The strength of this study lies in the active participation in RCs over time, which allows for a comprehensive understanding. Completing two comprehensive RC courses has provided an in-depth insight into facilitating co-creation and the nuanced significance of structural and cultural factors. The inclusion of facilitators with formal mental health training and those with experiential knowledge in focus groups enhances the richness and depth of the data, providing a well-rounded perspective.

Nonetheless, the findings of this study are based on a relatively small sample, as we conducted the study at only two sites. The small sample size may limit the generalisability of the results to a broader context and could affect the applicability of the findings to other settings. However, the detailed descriptions in the study enable readers to grasp the research context, methodologies, and conclusions thoroughly. Transparency aids in assessing the potential transferability of results to diverse fields.

## Conclusions

Co-creation processes in RCs transform facilitators’ roles by fostering equality and inclusiveness, which dissolves traditional service roles and strengthens social ties, creating a sense of communitas. These strengthened social ties enhance the sense of community and contribute significantly to the effectiveness of mental health interventions by providing a supportive network for facilitators and students.

The transformation of roles occurs in the tension between anti-structure and structure, necessitating humility and conscious judgments about disclosure from facilitators. Despite the positive transformations observed within RCs, achieving permanent changes in broader mental health services is challenging because of entrenched hierarchical structures and traditional role expectations. This resistance makes sustaining role transformations difficult when facilitators return to conventional settings. Understanding the interplay between anti-structure and structure is crucial, as these transformations are often temporary and may not integrate seamlessly into existing service frameworks.

Further research is needed to explore the sustainability of these transformed roles within mental health services, understand the integration of co-creation practices into broader settings, and examine the long-term effects on professional roles and service user outcomes. Future studies should focus on the scalability of these practices across different populations and settings to fully realise their potential benefits.

While the co-creation processes within RCs show promise in transforming roles and enhancing mental health interventions, considerable challenges remain in achieving and sustaining these changes across the broader mental health services landscape. Addressing these challenges will be crucial for developing more inclusive, effective, and responsive mental health care systems.

## Supplementary Information

Supplementary Material 1.

## Data Availability

The data collected for this study consisted of transcribed interviews and field notes. The readers can access part of the data supporting the study’s conclusions. We cannot release field notes and transcripts due to ethical restrictions. An interview guide (Additional file 1) is available.

## Abbreviations

RC: Recovery college
CHIME: Connectedness, hope, identity, meaning, and empowerment
Facilitator: When using the term facilitator, we refer to facilitators with formal mental health training and facilitators with experiential knowledge. If we are referring to facilitators with one of the backgrounds, we specify that by using the terms formal mental health training or experiential knowledge
