# Supplementary material for: Professional roles in transformation through sharing rituals: a critical ethnographic study of Norwegian Recovery Colleges
Source: BMC Health Serv Res. 2025 Mar 7;25:354. doi: 10.1186/s12913-025-12305-8 (PMC11889808; doi:10.1186/s12913-025-12305-8)
Supplement: Supplementary file 1 — Supplementary Material 1. [file 12913_2025_12305_MOESM1_ESM.docx]

Interview Guide for Facilitators

1. **Introduction**:
   - What is your affiliation with the recovery college?
   - What is your background?
   - What is your experience as a facilitator?
   - What is your current work affiliation?
2. **Co-creation**:
   - Co-creation involves people with lived experience working with professionals on course topics in the design and implementation of all parts of the college (Recollect).
     1. How is co-creation practised in the courses? Can you give examples?
     2. What is the relationship between experience and discipline in delivering courses?
     3. What aspects do the participants get to influence?
     4. How do you facilitate the participation of the participants in the course?
     5. How can you, as a facilitator, influence the course?
3. **Sharing Culture**:
   - How do you experience the sharing culture in the course?
     1. What are the benefits?
     2. What challenges have you encountered?
     3. What reflections do you have about what you share?
4. **Self-development**:
   - Have you experienced that participation in the Recovery College has led to self-development?
     1. In what way?
     2. What has contributed to this?
5. **Challenges**:
   - What factors inhibit the collaboration between course participants and course leaders in the Recovery College?
     1. What challenges have you faced with co-creation?
6. **Development**:
   - Do you think participating in recovery courses has influenced your work approach? How?
   - Has participation in co-creation processes in the course affected the development of the service where you are employed?
     1. How has it influenced interest in the workplace?
     2. How has it affected dialogue with the manager?
7. **Resistance**:
   - Have you experienced any resistance at your workplace?
     1. What do you think is the basis for this resistance?
     2. What have you done to deal with it?
     3. Has this led to more or less freedom to try new things and approaches?
